# Health Service Utilization in Hong Kong During the COVID-19 Pandemic – A Cross-sectional Public Survey

**DOI:** 10.34172/ijhpm.2020.183

**Published:** 2020-10-19

**Authors:** Kevin KC Hung, Joseph H. Walline, Emily Ying Yang Chan, Zhe Huang, Eugene Siu Kai Lo, Eng Kiong Yeoh, Colin A. Graham

**Affiliations:** ^1^Accident & Emergency Medicine Academic Unit, The Chinese University of Hong Kong, Prince of Wales Hospital, Hong Kong, China.; ^2^Collaborating Centre for Oxford University and CUHK for Disaster and Medical Humanitarian Response (CCOUC), The Chinese University of Hong Kong, Hong Kong, China.; ^3^JC School of Public Health and Primary Care, The Chinese University of Hong Kong, Hong Kong, China.; ^4^Nuffield Department of Medicine, University of Oxford, Oxford, UK

**Keywords:** Access to Healthcare, Health Seeking Behavior, Fear of Infection

## Abstract

**Background: **As health systems across the world respond to the coronavirus disease 2019 (COVID-19), there is rising concern that patients without COVID-19 are not receiving timely emergency care, resulting in avoidable deaths. This study examined patterns of self-reported health service utilization, their socio-demographic determinants and association with avoidable deaths during the COVID-19 outbreak.

**Methods:** A cross-sectional telephone survey was conducted between March 22 and April 1, 2020, during the peak rise in confirmed COVID-19 cases in Hong Kong. Cantonese-speaking Hong Kong residents over 18-years-old were recruited using a computerised random digital dialling (RDD) system. The RDD method used stratified random sampling to ensure a representative sample of the target population by age, gender, and residential district. A structured self-reported questionnaire was used.

**Results: **Out of 1738 placed calls, 765 subjects responded to the questionnaire (44.0% response rate). The factors associated with avoiding medical consultation included being female (37.2% vs. 22.5%, *P*<.001), married (32.8% vs. 27%, *P*=.044), completing tertiary education (35.3% vs. 27.7% (secondary) vs. 14.8% (primary), *P*=.005), and those who reported a "large/very large" impact of COVID-19 on their mental health (36.1% vs 30.5% (neutral) vs. 19.7% (very small/small),* P*=.047) using logistic regression analysis.

**Conclusion:** Married females with both higher educational attainment and concern about COVID-19 were associated with avoiding healthcare services. Timely public communication to encourage and promote early health seeking treatment even during extreme events such as pandemics are needed.

## Background

**Key Messages**

Implications for policy makers

- Reports from around the world have raised concerns about health service avoidance for critical illnesses during coronavirus disease 2019 (COVID-19).
- Married, more highly educated female respondents who reported a greater impact of COVID-19 on their mental health were associated with reporting avoidance of healthcare.
- Reduction in reported health service utilization was greatest for primary care clinics, emergency departments and general hospital admissions, in that order.
- Well-prepared health systems and timely communication are needed to overcome preventable morbidity and mortality during extreme events such as pandemics.

Implications for the public

During an infectious disease outbreak like coronavirus disease 2019 (COVID-19), it is important for the public to continue to have access to routine healthcare if possible. We found married, more highly educated female respondents who reported a greater impact of COVID-19 on their mental health were associated with avoiding medical care from this public survey in Hong Kong. Especially for patients suffering from medical emergencies like heart attacks or strokes, it is critical that emergency care is not delayed. Avoiding or delaying necessary medical care could lead to preventable deaths and disabilities.

As health systems across the world respond to the coronavirus disease 2019 (COVID-19), there is rising concern that patients with non-COVID-19 emergency conditions are avoiding care.^1-4^ In the Italian Paediatric Hospital Research Network, 12 cases of delayed access to hospital care were reported in 1 week, with 6 cases requiring admission to an intensive care unit and 4 deaths.^1^ Parents of these children reported avoiding hospitals for fear of contracting severe acute respiratory syndrome coronavirus 2 (SARS-CoV-2), the causative virus of COVID-19, with some even reporting being discouraged from attending a hospital by their usual healthcare providers. Healthcare providers as well as the general public appear to need clearer guidance and information on the relative risks of immediate versus delayed access to hospital emergency care.

 In England, there is also a rising concern that non-COVID-19 patients are not receiving timely emergency care, which may be leading to preventable deaths.^5^ The National Health Service (NHS) England reported a 29% fall in emergency department (ED) attendance and a 23% drop in emergency admissions in March 2020 compared to 2019, but a rise in registered non-COVID deaths. It is still unknown whether the drop in health service utilisation has impacted those who had time-critical medical emergencies and truly needed emergency care. The situation in the UK has exposed the difficulties of constructing a comprehensive and consistent picture of the wider effect of COVID-19 and the measures associated with controlling this pandemic.

 The Hong Kong Government activated the ‘Serious Response Level’ (the second of 3 alert levels) of the novel infectious disease plan on January 4, 2020 and identified the first 2 cases of COVID-19 in Hong Kong on January 22, 2020.^6^ Since then, statistics from the Hong Kong Hospital Authority have shown a territory-wide reduction in the number of patients attending the ED during COVID-19.^7^ ED attendances across Hong Kong plummeted 37% for the period from February through April year-on-year. Queen Mary Hospital, a university teaching hospital in Hong Kong, reported significant delays in presentation of ST-elevation myocardial infarction (STEMI)^2^ and acute stroke^8^ patients since the end of January. The study on STEMI patients also found delays in evaluation and treatment after hospital arrival, due to precautionary measures taken before transferring patients to the catheterisation laboratory and the application of full protective gear prior to commencing the procedure. Even though it is unclear whether such delays could lead to worse outcomes and preventable morbidity and mortality, it is important to understand the effect of COVID-19 on health service utilization by the general public including access to the ED.

 This study aimed to investigate the decreased utilization of health services by the Hong Kong public during the COVID-19 outbreak and identify factors associated with those avoiding medical care.

## Methods

 A cross-sectional, population-based landline telephone survey was conducted between March 22 and April 1, 2020, during the peak rise in confirmed COVID-19 cases in Hong Kong. This paper presents the health services utilization aspect of a public survey looking at the sociodemographic predictors of health risk perception, attitude and behaviour practices during the COVID-19 pandemic in Hong Kong, whose detailed methodology has been described elsewhere.^9^ In summary, the study population included all Cantonese-speaking Hong Kong residents over 18-years-old but excluded visitors holding tourist visas to Hong Kong. Sample size estimation was based on 50% of the Hong Kong population being concerned about contracting COVID-19 and found that 750 participants were needed to provide a 3.6% margin of error and a 95% confidence interval.

 Randomly generated telephone numbers from the list of all landline telephone numbers in Hong Kong were used as the sampling frame. Participants were recruited using a computerised random digital dialling (RDD) system. The RDD method uses stratified random sampling to ensure a representative sample of the target population by age, gender, and residential district. A structured self-reported questionnaire was used (Supplementary files 1 and 2) which included 6 major domains including sociodemographic information, knowledge about COVID-19, risk perception of behavioural measures associated with COVID-19, self-reported perceived usefulness and actual behavioural practices adopted, preferred channels of information acquisition, and home quarantine and caregiving to family members during outbreak. Each interview lasted about 20-40 minutes, and a pilot survey study was conducted in March 2020 to ensure question reliability.

 All statistical analyses were conducted using IBM SPSS Statistics for Windows, v25 (IBM Corp, Armonk, NY, USA). Descriptive statistics were reported for all participants, including those who avoided medical consultation. Percentages indicating avoidance were calculated based on an affirmative response to: ‘Have you avoided medical consultation since January 2020?’ divided by total participants. Missing responses were excluded from further analyses. Chi-squared tests were used for bivariate analysis. All variables were included in the binary logistic regression analyses to identify significant predictors of avoidance. Backward (Wald) method was used with probability for removal at 0.1 for each step. A value of *P*< .050 was considered to be statistically significant.

## Results

 Out of 1738 placed calls, 765 subjects responded to the questionnaire (44.0% response rate). Our sample was comparable to Hong Kong’s general population, but did have higher educational attainment and household income. 30.4% (232/764, 1 missing) reported avoidance of medical consultation due to COVID-19. Table 1 highlights participant characteristics. 99.6% of respondents were of Chinese ethnicity. Eleven respondents reported being tested for COVID-19 and 5 (0.7%) reported contracting COVID-19.

**Table 1:** Respondent Characteristics and Health Service Avoidance

	**All Respondents ** **(n = 765)**	**% Indicating Avoidance** ^a^ ** (n = 232)**	**Bivariate ** ***P *** **Values** ^b^	**Adjusted OR ** **(95% CI)**	**Multivariable Analysis** ^c^ ***P *** **Values**
Gender					
Male	356 (46.5%)	80 (22.5%)	<0.001	0.460 (0.323-0.655)	<0.001
Female	409 (53.5%)	152 (37.2%)		1	
Age group					
18-24	71 (9.3%)	21 (29.6%)	0.080		
25-44	248 (32.4%)	89 (35.9%)			
45-64	303 (39.6%)	88 (29.1%)			
65 or older	143 (18.7%)	34 (23.8%)			
Marital status					
Non-married	304 (39.9%)	82 (27.0%)	0.090	0.692 (0.483-0.991)	0.044
Married	459 (60.1%)	150 (32.8%)		1	
Education level					
Primary level or below	61 (8.0%)	9 (14.8%)	0.002	0.328 (0.149-0.721)	0.005
Secondary	330 (43.3%)	91 (27.7%)		0.651 (0.455-0.933)	
Tertiary level	371 (48.7%)	131 (35.3%)		1	
Profession					
White collar	341 (45.2%)	120 (35.2%)	0.040		
Blue collar (including services or sales)	128 (17.0%)	31 (24.2%)			
Homemaker	93 (12.3%)	30 (32.3%)			
Student	47 (6.2%)	15 (31.9%)			
Unemployed or retired	145 (19.2%)	33 (22.8%)			
Religion					
No religion	509 (67.2%)	150 (29.5%)	0.390		
Any	249 (32.8%)	81 (32.5%)			
Chronic disease					
No	624 (81.6%)	191 (30.7%)	0.713		
Yes	141 (18.4%)	41 (29.1%)			
Flu vaccine in the past 12 months					
No	560 (73.5%)	164 (29.3%)	0.252		
Yes	202 (26.5%)	68 (33.7%)			
Household income (monthly HK$)					
<2000-7999	66 (9.2%)	13 (19.7%)	0.135		
8000-19 999	100 (13.9%)	27 (26.7%)			
20 000-39 999	191 (26.6%)	65 (34.0%)			
40 000 or more	360 (50.2%)	114 (31.7%)			
Residential district					
Hong Kong Island	147 (19.2%)	57 (38.8%)	0.046	1.659 (1.059-2.599)	0.069
Kowloon	231 (30.2%)	67 (29.0%)		1.020 (0.688-1.512)	
New territories	387 (50.6%)	108 (28.0%)		1	
Housing type					
Public housing	219 (28.6%)	62 (28.4%)	0.054		
Subsidized housing	108 (14.2%)	24 (22.2%)			
Private housing	435 (57.2%)	146 (33.6%)			
Household members younger than 15 or older than 59					
No	264 (34.8%)	72 (27.3%)	0.179		
Yes	495 (65.2%)	158 (32.0%)			
Having a very high risk of contracting COVID-19 in year 2020					
Disagree or totally disagree	332 (44.1%)	86 (25.9%)	0.025		
Neutral	286 (38.0%)	103 (36.0%)			
Agree or totally agree	136 (17.9%)	41 (30.4%)			
Effect of COVID-19 on your physical health					
Small or very small effect	192 (25.3%)	39 (20.3%)	0.002	0.655 (0.398-1.078)	0.097
Neutral	173 (22.8%)	59 (34.1%)		1.167 (0.752-1.811)	
Large or very large effect	394 (51.8%)	133 (33.8%)		1	
Effect of COVID-19 on your mental health					
Small or very small effect	193 (25.3%)	38 (19.7%)	<0.001	0.524 (0.313-0.878)	0.047
Neutral	213 (27.9%)	65 (30.5%)		0.788 (0.514-1.178)	
Large or very large effect	358 (46.8%)	129 (36.1%)		1	
Enough knowledge to deal with COVID-19 outbreak to protect personal health					
Lacking or very lacking	95 (12.4%)	24 (25.3%)	0.015	0.962 (0.546-1.694)	0.082
Neutral	304 (39.7%)	110 (36.3%)		1.469 (1.020-2.116)	
Sufficient or very sufficient	366 (47.9%)	98 (26.8%)		1	

Abbreviations: OR, odds ratio; COVID-19, coronavirus disease 2019. ^a^ Based on the response to the question: ‘Have you avoided medical consultation since January 2020?’ (missing response to this question was excluded from subsequent analyses). ^b^ Chi square tests. ^c^ Binary logistic regression analysis including all listed variables.

 To summarize, after logistic regression analysis, female gender (*P* < .001), being married (*P* = .044), higher educational achievement (*P* = .005) and a larger mental health impact of COVID-19 (*P* = .047) were confirmed to be statistically significant (see Table 1 for complete results). The final model included 7 variables including gender, marital status, residential district, effect of COVID-19 on physical health, effect of COVID-19 on mental health, and enough knowledge to deal with COVID-19 outbreak to protect personal health, with good reliability (Hosmer-Lemeshow test *P* = .856) in the final model.

 Respondents also reported varying frequencies of healthcare utilization (Table 2), with more respondents reporting reduced visits to private general practitioners (31.2%), followed by public primary care clinics (26.1%), EDs (25.2%), public hospital admissions (17.3%) or private hospital admissions (14.8%).

**Table 2:** Reported Changes to Utilisation of Various Healthcare Services

	**More**	**No Difference**	**Less**
Visiting private general practitioners	21 (2.7%)	505 (66.1%)	238 (31.2%)
Visiting public general out-patient clinics	17 (2.2%)	546 (71.7%)	199 (26.1%)
Visiting hospital EDs	17 (2.2%)	554 (72.6%)	192 (25.2%)
Admission to public hospitals (if advised by a physician)	21 (2.7%)	611 (80.0%)	132 (17.3%)
Admission to private hospitals (if advised by a physician)	23 (3.0%)	629 (82.2%)	113 (14.8%)

Abbreviation: ED, emergency department.

 Based on data from the Hong Kong Census (2018-2019), the most common medical consultation pattern by Hong Kong residents was to attend private general practitioners, followed by public general practitioners (including general outpatient clinics) and then attendance at hospital EDs.^10^

## Discussion

 Our study found 30.4% of the public reported avoiding medical consultations during the COVID-19 outbreak in Hong Kong. We identified several respondent characteristics such as female gender, marriage status, higher educational attainment, as well as the mental health effects of COVID-19 as significant factors associated with health service avoidance.

 Similar to our findings from Hong Kong, studies from Italy and the United States also showed a reduction of 48.4% in cases of acute myocardial infarction^3^ and a reduction of 38% in admissions for STEMI.^4^ In Italy, De Rosa et al found that this reduction was greater in women than men (41.2% vs, 17.8%),^3^ however they did not look at marriage status or educational level in their study. Both patient and system related delays were also observed: time from symptom onset to coronary angiography increased by 39.2%, time from first medical contact to coronary angiography increased by 31.5%, and the STEMI case fatality rate increased (relative risk 3.3) during COVID-19.^3^

 Possible reasons for avoiding necessary medical care may include fear of contracting COVID-19, as was previously found during the severe acute respiratory syndrome (SARS) outbreak of 2003.^11^ Middle East respiratory syndrome (MERS) and SARS both had high levels of transmission in healthcare settings,^12^ the memory of which may have reduced health service utilisation during COVID-19. During SARS, we saw reduced ED attendance for trauma or minor care, but not for those requiring immediate care.^13^ Prince of Wales Hospital, a large, tertiary-care teaching hospital in northeast Hong Kong, was the site of a significant SARS outbreak among patients and staff in March 2003, and the hospital’s ED had to be closed during the SARS epidemic to concentrate hospital resources on containing the disease. Subsequently, as the ED reopened and resumed service in April 2003, the daily ED attendance dropped by more than half (265 vs. 545) in April compared with 2002. Trauma cases fell by 64% (40 vs. 111) and minor cases were nearly halved by 54% (193 vs. 421). These changes were attributed to community behaviour in reducing social contact and avoiding hospitals in general for fear of contracting SARS, and especially those hospitals like Prince of Wales that were associated with nosocomial outbreaks. Out of a sense of altruism, people may also not want to add pressure to an already strained healthcare system.

 During a pandemic, it is important for healthcare systems to ensure access to safe and reliable care, especially for those with chronic diseases. Although there was no statistically significant difference between participants with and without chronic diseases, it is concerning that 29.1% of respondents with a self-reported chronic disease also indicated avoidance, which may have significant repercussions on routine care. Wolf et al recently reported that since the COVID-19 outbreak in the United States began, adults with chronic conditions lacked critical knowledge about COVID-19.^14^ Black race and low heath literacy were both independently associated with a greater likelihood of feeling less prepared for COVID-19. The World Health Organization (WHO) acknowledged that people living with non-communicable diseases (NCDs) are more vulnerable to both becoming severely ill and dying from COVID-19 and there may be a long-term upsurge in deaths from NCDs due to disruption of NCD services.^15^ Results from their recent rapid assessment from 163 countries in May 2020 showed that cancellation of elective care (65% of countries), closure of population level screening programmes (46%), and lockdowns hindering access to health facilities (43%) were major reasons for NCD service disruption. Decreased outpatient volumes due to patients not presenting was observed in 25% of responding countries. Telemedicine deployment to replace in-person consultations, and triaging to identify priorities were the mitigation strategies most often used to overcome disruptions.

 In fact, telemedicine has been suggested as one solution to this problem early in this COVID-19 pandemic.^16^ In the Netherlands, telemedicine was quickly adopted in the hardest hit COVID-19 region for general primary care visits and for those patients with chronic diseases.^17^ The reduction in the number of physical consultations was matched by a comparable increase in e-consultations and telephone consultations in the Netherlands, and total consultation numbers actually increased by week 14 of the pandemic after an initial drop.

## Limitations

 This study focused on the public’s self-perception of healthcare avoidance, and it is uncertain to what degree actual medical consultations changed in Hong Kong. However, the 30% reported reduction in health service utilization we found in this study is close to the confirmed 37% reduction in ED attendance as recorded by the 18 public hospitals in Hong Kong during the same period. Although we found statistically significant associations between reported reductions in healthcare utilization and married, more highly educated women who seemed to fear COVID-19 more, further research is needed to identify in more detail why these associations exist and what elements of these associations or their interactions led to less healthcare utilization in during COVID-19.

 Secondly, although our sample was generally representative of the Hong Kong population, there may have been some sampling bias resulting from the modest response rate of 44%. Households without landline telephone service may have been missed, even though the penetration rate of landline telephones reached 85.5% in 2019.^18^

 Third, the data collection period coincided with the sharpest rise in the number of confirmed COVID-19 cases (492 out of the total 1066 confirmed cases as of May 24, 2020 were confirmed during the study period).^19^ New public health measures including a government prohibition of group gatherings of more than 4 people in public places were introduced during the study period. This may have had an unintended impact on the participants’ response to COVID-19, possibly introducing recall bias due to the heightened public response.

 Lastly, the COVID-19 outbreak and the public health response in Hong Kong is different from other regions across the world. The findings may not be directly generalizable to other regions.

## Conclusion

 We found that a significant minority of the Hong Kong public reported a reduction in health service utilization during COVID-19, with the greatest reduction occurring in visits to primary care clinics followed by ED visits and hospital admissions. It is important for health systems to ensure access to safe and reliable healthcare for non-COVID patients, especially for those with emergent or chronic diseases. It is essential that adequate public messaging and communications are maintained during the pandemic, so that life threatening emergencies are not delayed. Well-prepared EDs and resilient healthcare systems, along with timely communication with the public, are needed to protect our patients.

## Ethical issues

 Survey and Behavioural Research Ethics Committee at The Chinese University of Hong Kong approved the study (SBRE-19-498). Verbal consent was obtained from all participants.

## Competing interests

 Authors declare that they have no competing interests.

## Authors’ contributions

 KKH, EYYC conceived and designed the study. ZH, ESKL, and EYYC conducted the study and performed data collection. ZH, ESKL, and EYYC managed the data, including quality control. KKH, ZH, ESKL, EKY, JHW, and CAG provided statistical advice on study design and analyzed the data. KKH, JHW drafted the manuscript, and all authors contributed substantially to its revision. All authors agreed to the final approval of the version to be published. All authors agreed to be accountable for all aspects of the work in ensuring that questions related to the accuracy or integrity of any part of the work are appropriately investigated and resolved.

## Authors’ affiliations

^1^Accident & Emergency Medicine Academic Unit, The Chinese University of Hong Kong, Prince of Wales Hospital, Hong Kong, China. ^2^Collaborating Centre for Oxford University and CUHK for Disaster and Medical Humanitarian Response (CCOUC), The Chinese University of Hong Kong, Hong Kong, China. ^3^JC School of Public Health and Primary Care, The Chinese University of Hong Kong, Hong Kong, China. ^4^Nuffield Department of Medicine, University of Oxford, Oxford, UK

## Supplementary files


Supplementary file 1. Summary of Full Questionnaire.

Supplementary file 2. Questions From Questionnaire Used in the Current Study.

## References

[1] Lazzerini M, Barbi E, Apicella A, Marchetti F, Cardinale F, Trobia G (2020). Delayed access or provision of care in Italy resulting from fear of COVID-19. Lancet Child Adolesc Health.

[2] Tam CF, Cheung KS, Lam S (2020). Impact of coronavirus disease 2019 (COVID-19) outbreak on ST-segment-elevation myocardial infarction care in Hong Kong, China. Circ Cardiovasc Qual Outcomes.

[3] De Rosa S, Spaccarotella C, Basso C (2020). Reduction of hospitalizations for myocardial infarction in Italy in the COVID-19 era. Eur Heart J.

[4] Garcia S, Albaghdadi MS, Meraj PM (2020). Reduction in ST-segment elevation cardiac catheterization laboratory activations in the United States during COVID-19 pandemic. J Am Coll Cardiol.

[5] Appleby J (2020). What is happening to non-covid deaths?. BMJ.

[6] Hung KKC, Walline JH, Graham CA (2020). COVID-19: emergency medicine perspectives from Hong Kong. Eur J Emerg Med.

[7] Hospital Authority Clinical Data Analysis and Reporting System, Hospital Authority, Hong Kong SAR. Accessed May 7, 2020.

[8] LKS Faculty of Medicine, The University of Hong Kong. News: HKUMed Research Shows that Stroke Patients are Presenting to Hospitals One Hour Later During COVID-19, Potentially Jeopardising the Patients’ Eligibility for Treatments and Affecting the Outcome. HKUMed; 2020. https://www.med.hku.hk/en/News/stroke-patients-are-presenting-to-hospitals-one-hour-later-during-COVID-19. Accessed May 27, 2020.

[9] Chan EYY, Huang Z, Lo ESK, Hung KKC, Wong ELY, Wong SYS (2020). Sociodemographic predictors of health risk perception, attitude and behavior practices associated with health-emergency disaster risk management for biological hazards: the case of COVID-19 pandemic in Hong Kong, SAR China. Int J Environ Res Public Health.

[10] Census and Statistics Department, Hong Kong Special Administrative Region. Thematic Household Survey Report No. 68. https://www.statistics.gov.hk/pub/B11302682019XXXXB0100.pdf. Accessed May 11, 2020.

[11] Chang HJ, Huang N, Lee CH, Hsu YJ, Hsieh CJ, Chou YJ (2004). The impact of the SARS epidemic on the utilization of medical services: SARS and the fear of SARS. Am J Public Health.

[12] Chowell G, Abdirizak F, Lee S (2015). Transmission characteristics of MERS and SARS in the healthcare setting: a comparative study. BMC Med.

[13] Man CY, Yeung RS, Chung JY, Cameron PA (2003). Impact of SARS on an emergency department in Hong Kong. Emerg Med (Fremantle).

[14] Wolf MS, Serper M, Opsasnick L (2020). Awareness, attitudes, and actions related to COVID-19 among adults with chronic conditions at the onset of the US outbreak: a cross-sectional survey. Ann Intern Med.

[15] World Health Organization (WHO). Rapid Assessment of Service Delivery for NCDs During COVID-19 Pandemic. Geneva: WHO; 2020.

[16] Hollander JE, Carr BG (2020). Virtually perfect? Telemedicine for Covid-19. N Engl J Med.

[17] Auener S, Kroon D, Wackers E, Dulmen SV, Jeurissen P (2020). COVID-19: a window of opportunity for positive healthcare reforms. Int J Health Policy Manag.

[18] Office of the Communications Authority. Key Communications Statistics. Hong Kong Special Administrative Region. https://www.ofca.gov.hk/en/data_statistics/data_statistics/key_stat/. Accessed May 27, 2020.

[19] Centre for Health Protection, Department of Health, Hong Kong Special Administrative Region. Latest situation of cases of COVID-19 (as of May 24, 2020). https://www.chp.gov.hk/files/pdf/local_situation_covid19_en.pdf. Accessed May 25, 2020.

