# Supplementary material for: Health Service Utilization in Hong Kong During the COVID-19 Pandemic – A Cross-sectional Public Survey
Source: Int J Health Policy Manag. 2020 Oct 19;11(4):508–13. doi: 10.34172/ijhpm.2020.183 (PMC9309937; doi:10.34172/ijhpm.2020.183)
Supplement: Supplementary file 1 — Summary of Full Questionnaire. [file ijhpm-11-508-s001.pdf]

Supplementary 1. Summary of Full Questionnaire

1. Sociodemographic and background information of the participants, including age group, gender, area of residence, household income, household size, marital status, education attainment, size of housing, occupation and employment status.
2. Knowledge and risk perception of coronavirus disease 2019 (COVID-19), including the transmission routes, the perceived impacts (eg, physical, mental, social, financial and the whole impact), perceived sufficient knowledge to manage COVID-19, perceived severity and infectivity. The 6-item short form of the State-Trait Anxiety Inventory (STAI) was used for measuring the anxiety about the current outbreak. A question of whether the respondent worried about getting infected with COVID-19 was asked.
3. Behavioral patterns during COVID-19, including health service utilization and household preparation of medical supplies.
4. Self-reported perceived usefulness and actual practice of some preventive measures against COVID-19, including (1) washing hands before meals and after toileting, (2) washing hands with soaps, (3) avoiding dining or gathering together, (4) using serving utensils, (5) ordering takeaways more often, (6) bringing one's own utensil when dining out, (7) wearing a mask when going out, (8) avoiding going to public places or using public transport, and (9) avoiding going to COVID-19-confirmed regions outside Hong Kong.
5. Current and preferred channels of COVID-19 related information acquisition, the information they were interested in and the awareness of COVID-19.
6. Questions about home quarantine and caregiving to non-suspected family members during the COVID-19 outbreak.
