# Supplementary material for: Health Service Utilization in Hong Kong During the COVID-19 Pandemic – A Cross-sectional Public Survey
Source: Int J Health Policy Manag. 2020 Oct 19;11(4):508–13. doi: 10.34172/ijhpm.2020.183 (PMC9309937; doi:10.34172/ijhpm.2020.183)
Supplement: Supplementary file 2 — Questions From Questionnaire Used in the Current Study. [file ijhpm-11-508-s002.pdf]

Supplementary file 2. Questions From Questionnaire Used in the Current Study

1. **Gender**

1. Male 2. Female

2. **What is your age?**

1.	18-19	2.	20-24	3.	25-29	4.	30-34
5.	35-39	6.	40-44	7.	45-49	8.	50-54
9.	55-59	10.	60-64	11.	65-69	12.	≥70

999. Refuse to answer

3. **Do you agree that the COVID-19 virus testing should be available for everyone who is worried of getting the disease? (read option)**

1. Totally disagree 2. Disagree 3. Neutral 4. Agree
5.Totally agree 888. Don't know 999. Refuse to answer

4. **Have you been diagnosed with COVID-19?**

1) No 2) Yes 3) Don't know 4) 999.Refuse to answer

5. **Has anyone in close proximity to you been diagnosed with COVID-19? (The close proximity is defined as living in the same household, living in the same building does not count)**

1) No 2) Yes 3) Don't know 4) 999.Refuse to answer

Here I will ask few questions about COVID-19. Please give a score from 1-5 in the following questions (Please explain what score 1 and 5 mean when asking the question):								**Don't know**	Refuse
**6. The chance of being infected by COVID-19 this year is very high (If already 4a answer yes, code=6)?**	*Totally disagree*	*1*	*2*	*3*	*4*	*5*	*Totally agree*	888	999
**7. How much has COVID-19 impacted your health？**	*No impact at all*	*1*	*2*	*3*	*4*	*5*	*Very high impact*	888	999
**8. How much has COVID-19 impacted your mental？**	*No impact at all*	*1*	*2*	*3*	*4*	*5*	*Very high impact*	888	999
**9. Do you have sufficient knowledge to manage the risks to health and personal safety during the COVID-19 outbreak?**	*Totally insufficient*	*1*	*2*	*3*	*4*	*5*	*Very sufficient*	888	999

10. **Have you used more or less the health services during COVID-19 epidemic (read each option below)**

i. Private doctor

1. more likely 2. same 3.less likely 888.Don't know 999. Refuse

ii. Public government clinic

1. more likely 2. same 3.less likely 888.Don't know 999. Refuse

iii. A&E

1. more likely 2. same 3.less likely 888.Don't know 999. Refuse

iv. Admission to public hospital (if advised by doctor)

1. more likely 2. same 3.less likely 888.Don't know 999. Refuse

v. Admission to private hospital (if advised)

1. more likely 2. same 3.less likely 888.Don't know 999. Refuse

11. Since January, have you avoided going to a doctor because of COVID-19?

1.No 2.Yes 888.Don't know 999.Refuse to answer

12. Do you have any form of chronic disease?

1. No 2. Yes 888.Don't know 999.Refuse to answer

13. What is your marital status?

1. Never married 2. Now married 3. Widowed 4. Divorced
5. Separated 888.Don't know 999.Refuse to answer

14. What is your highest education level obtained?

1. No schooling/pre-primary 2. Primary 3. Lower secondary
4. Form 5 graduate 5. Upper secondary/graduated secondary school
6. Diploma/certificate 7. Higher diploma 8. Associate's degree
9. Bachelor's Degree 10. Master's or above
11. Others (please specify) ______________________ 888.Don't know 999.Refuse to answer

15. What is your occupation?

1. Managers and administrators 2. Professionals 3. Associate professionals
4. Clerks 5. Service workers and shop sales workers 6. Craft and related workers
7. Plant and machine operators and assemblers 8. Elementary occupations
9. Skilled agricultural and fishery workers 10. Housewives 11. Student
12. Retired 13. Unemployed 14. Others (please specify) _______________

888.Don't know 999.Refuse to answer

16. Have you received a Flu vaccine within the past 12 months?

1 No 2 Yes 3 Cannot remember

888 Don't know 999 Refuse

17. What is your religious affiliation ? (Don't need to read out, can only choose 1)

1. No religious belief 2. Christianity 3. Catholism 4. Buddhism
5. Taoism 6. Islam 7. Worship Chinese Gods \ ancestors at home
8. Others: ____________ 888.Don't know 999.Refuse to answer

18. What type of housing do you live in?

1. Public housing 2. House Ownership Scheme 3. Private estates
4. Wooden house/temporary shelter 5. Private house (village house or mansions)
6. Sublet rooms 7. Cubicle or bed-space 888. Don't know 999. Refuse to answer
8. Others (please specify) ____________

19. Including you, are there any member in your household who is under 15 years old or older than 59 years old?

1. No 2. Yes 888. Don’t know 999. Refuse to answer

20. What is your monthly household income (HKD)?

1.	< 2,000	2.	2,000 - 3,999	3.	4,000 - 5,999	4.	6,000 - 7,999
5.	8,000 - 9,999	6.	10,000 - 14,999	7.	15,000 - 19,999	8.	20,000 - 24,999
9.	25,000 - 29,999	10.	30,000 - 39,999	11.	40,000 - 59,999	12.	≥ 60,000
888.Don’t know		999.Refuse to answer					

21. Which district do you live in?

1.	Central & Western	2.	Wan Chai	3.	Eastern	4.	Southern
5.	YauTsimMong	6.	Sham Shui Po	7.	Kowloon City	8.	Wong Tai Sin
9.	Kwun Tong	10.	KwaiTsing	11.	Tsuen Wan	12.	TuenMun
13.	Yuen Long	14.	North	15.	Tai Po	16.	Sha Tin
17.	Sai Kung	18.	Islands	888.Don’t know		999.Refuse to answer	
